# Multiple resistance-activating substances produced by *Humicola phialophoroides* isolated from soil for control of Phytophthora blight of pepper

**DOI:** 10.1186/1999-3110-55-40

**Published:** 2014-09-11

**Authors:** Ching-Hui Yang, Mei-Ju Lin, Huey-Jen Su, Wen-Hsiung Ko

**Affiliations:** 1 Department of Plant Pathology, National Chung Hsing University, Taichung, Taiwan; 2 Department of Nursing, Meiho University, Neipu, Pingtung Taiwan

**Keywords:** *Humicola phialophoroides*, Pepper, *Phytophthora capsici*, Resistance activation

## Abstract

**Background:**

Microorganisms capable of utilizing vegetable tissues for multiplication in soil were isolated, cultivated in liquid medium prepared from the same vegetable tissues, and tested for ability to activate resistance in pepper leaves against Phytophthora blight caused by *Phytophthora capsici*.

**Results:**

Among the 121 microorganisms isolated, a fungus *Humicola phialophoroides* showed distinct ability to produce substances capable of activating resistance. The resistance-activating substances produced by *H. phialophoroides* were mostly retained in the mycelium, and were readily extracted from the mycelium powder with polar solvents. The extract was not inhibitory to zoospore germination or germ tube growth of *P. capsici*. In pepper leaves, the extract took only about 12 h to activate resistance against *P. capsici*. After activation, washing treated leaf surface with water did not have much effect on the resistance expression. In addition to being able to move from the upper leaf surface to lower leaf surface, the resistance-activating substances were capable of moving 5 mm acropetally and 10 mm basipetally in pepper leaves, Chromatography of the extract on silica gel column suggests that there are probably more than three components in the extract with resistance-activating ability. The resistance-activating activity of the mycelium extract was not affected by treatment with either cation or anion exchange resins, indicating that none of the active components have positive or negative charges on their molecules.

**Conclusion:**

Results show that *H. phialophoroides* is capable of producing multiple resistance-activating substances which are mostly retained in the mycelium. The study also indicates that none of the active components have positive or negative charges on their molecules.

**Electronic supplementary material:**

The online version of this article (doi:10.1186/1999-3110-55-40) contains supplementary material, which is available to authorized users.

## Background

Phytophthora blight caused by *Phytophthora capsici* is a devastating disease of pepper (*Capsicum annuum* L.) and several other economically important crops (Hausbeck and Lamour,^2004^ ; Hwang and Kim,^1995^). The disease is of worldwide importance and causes significant economic losses each year. Fungicides have been commonly used for the control of Phytophthora blight of pepper (Hwang and Kim,^1995^). However, alternative methods of disease control are needed because of the emergence of pathogen strains resistant to the chemicals such as mefenoxam and metalaxyl (Parra and Ristaino,^2001^) and the high concern about the adverse effects of pesticides on human health and the environment (World Health Organization,^1990^).

A commonly recommended method for plant disease control is the use of resistant cultivars. Very few commercial pepper cultivars with strong resistance to *P. capsici* are available (Hausbeck and Lamour,^2004^; Hwang and Kim,^1995^). Moreover, resistance is affected by environment and the appearance of virulent pathogen strains (Hausbeck and Lamour,^2004^). Consequentially, resistance activation has emerged as an important alternative in the control of oomycete and fungal plant pathogens (Barbosa et al.,^2008^). Microorganisms have been considered to be a possible source of potentially novel resistance activators (Lyon and Newton,^1999^). Since soil contains abundant and diverse microorganisms (Alexander,^1997^), it was hypothesized to be an ideal place to search for microorganisms capable of producing resistance-activating substances.

Vegetable tissues are rich in nutrients for growth and production of antimicrobial compounds in soil useful for management of soilborne plant pathogens (Gamliel et al.,^2000^). It was, therefore, considered possible to use vegetable tissues as nutrients for soil microorganisms for production of resistance-activating substances for control of plant diseases.

The objective of this study was to develop a technique that can be used to screen for soil microorganisms capable of utilizing vegetable tissues as nutrients for growth and production of resistance-activating substances for control of Phytophthora blight of pepper. The nature of the resistance-activating substances produced by *Humicola phialophoroides*, the soil microorganism obtained with this technique is also reported herein.

## Methods

### Isolation and cultivation of soil microorganisms

Soil samples collected from four different locations in Kaohsiung, Taiwan were taken from a depth of 0–10 cm, sifted and moistened to about 65% water-holding capacity. Microorganisms capable of utilizing vegetable tissues, consisting of small pieces of leaves and stems of spinach (*Spinacia oleracea* L.), ong choy (*Ipomoea aquatica* Forsskal) and common purslane (*Portulaca oleracea* L.), fruit of tomato (*Lycopersicon esculentum* Mill.), and tubers of sweet potato [*Ipomoea batatas* (L.) Lam.], for multiplication in soil were isolated as previously described (Ko et al.,2010b).

For cultivation of isolated microorganisms, vegetable broth was prepared by grinding 4 g each of the vegetables mentioned above in 100 ml water in a juice blender for 2 min and dispensing 50 ml broth in a 250-ml flask. After autoclaving, each flask was inoculated with two loopfuls of bacteria or a piece (4 × 5 × 3 mm) of actinomycete or fungus agar culture. Inoculated flasks were incubated on a shaker at 100 strokes/min for 2 weeks. After incubation, cultures were separately ground in a juice blender for 2 min before being used to test their ability to activate resistance.

### Single spore isolation

Single-conidium isolates were obtained by shaking a culture block (5 × 8 × 3 mm) of isolate KVF-2, grown on 10% V8 agar (10% V8 juice, 0.02% CaCO_3_ and 2% agar) (Ko et al.,2010a) at 24°C under light for 12 days, in 1 ml sterile distilled water in a test tube on a Vortex Mixer for 30 s to suspend conidia in water. A 100-μl portion of condial suspension was evenly spread on 2% water agar plate, and after incubation at 24°C for 24 h, colonies originating from single conidium were individually transferred to V8 agar plates. Single-chlamydospore isolates were obtained by transferring 10 culture blocks (8 × 8 × 3 mm) from a 6-day-old culture of KVF-2 into 200 ml sterile distilled water in a 250-ml flask. After incubation at 24°C in darkness for 20 days to induce chlamydospore formation, mycelial mats with chlamydospores were triturated in an Omni Mixer at 4,000 rpm for 1 min. The suspension was passed through a 70-μm sieve to remove mycelial fragments, and 100 μl of the resulting filtrate was spread on a 2% water agar plate. After incubation at 24°C for 24 h, colonies originating from single chlamydospores were individually transferred to V8 agar plates.

### Preparation of inoculum

*Phytophthora capsici* (isolate 27172) was grown on V8 agar at 24°C for 5–7 days. A piece of culture block (10 × 15 × 3 mm) obtained near the center of the colony was placed in 15 ml sterile distilled water in a 6-cm plate, and transferred to 4°C for 30 min to induce zoospore release from sporangia. The zoospore suspension was pipetted into a test tube and adjusted to 5 zoospores/μl with a Pipetman microliter pipette (West Coast Scientific, CA, USA) (Ann et al.,^2010^ ; Ko et al.,^1973^).

### Assay of liquid cultures for activation of disease resistance

Seeds of bell pepper (*C. annuum* cv. California Wonder) were sown in 8-cm pots containing a mixture of perlite, vermiculite and peat moss (1:1:3, v/v). Four leaves of a 1-month-old plant were sprayed once to runoff with a culture of a test organism daily for 3 days before inoculation on the 4th day unless otherwise stated. Two leaves were inoculated on the upper sprayed surface with five 2-μl drops of a zoospore suspension of *P. capsici* along the edge of the leaf. A 10-μl drop of molten 1% agar and 1% V8 juice at 60°C was added to each inoculum drop to fix the inoculum on the target site (Ko et al.,2010c). The other two leaves were similarly inoculated on the lower unsprayed surface to test the ability to activate disease resistance. Leaves sprayed with liquid medium or distilled water were similarly inoculated and used as controls. Inoculated plants were placed in moist chambers and kept in the greenhouse. The number and size of lesions that developed at the inoculated sites were recorded 2 days after inoculation.

For detection of resistance-activating substances in the extract, 10 μl extract containing 0.1% Tween 80 was spread on each of five test circles (ca. 10 mm in diameter) on each leaf with a glass rod. Extract was applied three times within 1 h, and inoculation was performed on each circle after 24 h as described above. Two leaves were used for each treatment and all the experiments were carried out twice.

### Detection of the resistance-activating substances inside and outside the mycelium

To determine if the resistance-activating substances were in the liquid culture secreted from the mycelium or inside the mycelium, 25 ml of culture suspension was centrifuged at 3000 × *g* for 10 min. The supernatant was saved in a flask. The mycelial mat in the pellet was resuspended in 25 ml sterile distilled water and centrifuged, and the supernatant was discarded to remove residual liquid culture. The washed mycelial mat was ground in liquid nitrogen with a mortar and pestle and the powder was suspended in 25 ml sterile distilled water. It was also suspended in 25 ml sterile distilled water and subjected to ultrasonification (Ultrasonic Processor, Sonic, CT, USA) for 30 min before use in the bioassay.

### Extraction of the resistance-activating substances

To extract the resistance- activating substances, the mycelium pellet from 50 ml of culture was ground with liquid nitrogen as described above and the resulting powder was extracted with 50 ml of water, 50% ethanol, 95% ethanol, methanol, acetone or hexane in a 250-ml flask by shaking on a shaker for 24 h. The extract was filtered through a Whatman no. 1 filter paper, and the filtrate was evaporated to 25 ml followed by addition of 25 ml water and evaporation to 25 ml again. The extract was then adjusted to 50 ml with water before use in the bioassay.

### Characterization of the resistance-activating substances

To test the effect of the resistance-activating substances on spore germination, 10 μl of zoospore suspension (1 × 10^5^ spores/ml) of *P. capsici* containing 0.01% glucose was mixed with 10 μl of mycelium extract in a cavity of a sterile eight-cavity slide. Slides with spores were kept moist by placing each on an L-shaped glass rod in a 9-cm petri plate containing 10 ml sterile distilled water. Germination of zoospores and germ tube length were recorded after incubation at 28°C for 6 h. In each of the three replicates, 100 spores were counted and 100 germ tubes were measured.

Spore germination was also tested on leaves. Leaves were treated with mycelium extract three times in 1 h, and a 2-μl drop of zoospore suspension was placed on each test circle 24 h later. After 6 h in a moist chamber, the test circles were stained with rose bengal, and spore germination and germ tube length were determined under a microscope equipped with a vertical illuminator (Ko,^1971^).

To study the effect of pH on the resistance-activating activity of mycelial extract, the pH value was adjusted from the original 6.7 to 4 with 1 N HCl or to 10 with 1 N KOH. To determine the stability of the resistance- activating substances under high and low pH, a 2-fold high concentration of the mycelium extract was adjusted to pH 2 with 1 N HCl or to 12 with 1 N KOH. After 24 h, the pH of the extract was adjusted back to the original 6.7, and sterile distilled water at pH 6.7 was used to adjust the extract to the original concentration. The extracts were then tested on pepper leaves for the ability to activate resistance against *P. capsici* as described above.

To study the ability of different adsorptive materials to remove the resistance- activating substances from the mycelium extract, Diaion SK1B cation exchange resins (equivalent to Amberlite 1R-120), Diaion SA 12A anion exchange resins (equivalent to Amberlite 1RA-420) and activated charcoal (Sigma–Aldrich, St. Louis, MO, USA) were washed as previously described to remove possible inhibitory substances (Ko et al.,2010b). An aliquot of 10 ml extract was shaken with 1 g cation exchange resins, anion exchange resins or activated charcoal in a 150-ml flask on a shaker for 24 h and filtered through a Whatman no. 1 filter paper. The filtrates were then used for bioassay.

To determine if single or multiple components in the mycelium extract are involved in the resistance activation, 35 g mycelium powder from 5 liters of culture of *H. phialophoroides* was extracted with 50% ethanol. The resultant extract (8 g) was chromatographed on a silica gel column (70–230 mesh, 2.5 cm × 60 cm) eluted with n-hexane/acetone (8:2, v/v) to produce five fractions (fractions 1 to 5). Each fraction was subjected to bioassay after evaporation of solvents as described above.

### Data analysis

Results from individual experiments were subjected to ANOVA. Means of treatments were separated by Fisher’s protected least significant difference test at P = 0.05. Analyses were conducted with SPSS software (SPSS 6.1.3 for Windows). For each experiment, 10 replicates were used. All experiments were performed twice with similar results, and data from one of the experiments were presented.

## Results

### Activation of disease resistance by isolated microorganisms

From the four soil samples, 38 bacteria, 26 actinomycetes and 57 fungi which were capable of utilizing vegetable tissues for multiplication in soil were isolated. When sprayed on pepper leaves, most of the liquid cultures of isolated microorganisms did not have the ability to reduce disease incidence. However, the cultures of four fungi were able to reduce infection rate and lesion size caused by *P. capsici* (Table 1). Liquid culture of isolate KVF-2 was most effective, capable of reducing infection rate from 100% in the control to 20% and lesion diameter from 11 mm to 3 mm on the treated surface. Moreover, even on the back of the treated surface the lesion diameter was reduced from 12 mm in the control to 4 mm, indicating ability of this liquid culture to activate disease resistance on the untreated lower leaf surface. Liquid cultures of isolates KVF-5 and KVF-6 also reduced the lesion size to 7 to 8 mm in diameter on untreated lower leaves, indicating slight ability to activate host resistance. Isolate KVF-2 was identified as *Humicola phialophoroides* (Ko et al.,^2011^), and was selected for further study.

**Table 1:** **Effect of four liquid cultures of soil fungi from the first selection on incidence of Phytophthora blight of pepper caused by** ***Phytophthora capsici*** ^a^

Isolate	Inoculation site^b^			
	Upper leaf surface (treated surface)		Lower leaf surface (non-treated surface)	
	Infection rate (%)	Lesion size (mm)	Infection rate (%)	Lesion size (mm)
KVF-1	50	6 b	100	12 a
KVF-2	20	3 c	90	4 c
KVF-5	70	7 b	100	8 b
KVF-6	70	7 b	90	7 b
Medium (control)	100	11 a	100	12 a

^a^Four leaves were treated with a liquid culture on the upper side daily for 3 days before inoculation on the 4th day on two upper leaves and two lower leaves.

^b^Values followed by the same letter in the same column are not significantly different using Fisher’s least significant difference test at *P* = 0.05.

### Selection of isolate with strong resistance-activating ability

Single-conidium and single-chlamydospore isolates of *H. phialophoroides* were each cultivated in liquid medium and the cultures were tested for resistance activation ability as described above. On the treated upper leaf surface, the three single-conidium isolates were not better than the original isolate in reducing the infection rate and lesion size (Table 2). However, on the untreated lower leaf surface, the culture of isolate S2 was able to reduce the lesion size more than 50% compared to leaves treated with the culture of the original isolate, indicating better resistance activation ability. Among the 10 cultures of single-chlamydospore isolates, six were able to prevent the disease development on the treated upper leaf surface just like the original isolate culture, and four of them were able to reduce the lesion size on the untreated lower leaves more than 50% of leaves treated with the original isolate culture. The culture of isolate B6 was especially active, reducing the lesion diameter from 9 mm treated with the original isolate culture to 1 mm. Isolate B6 was, therefore, selected for subsequent study.

**Table 2:** **Comparison of ability of liquid cultures of single-spore isolates of** ***Humicola phialophoroides*** **KVF-2 to reduce disease incudence of Phytophthora blight of pepper caused by** ***Phytophthora capsici*** ^**a**^

Origin and isolate	Inoculation site^b^			
	Upper leaf surface (treated surface)		Lower leaf surface (non-treated surface)	
	Infection rate (%)	Lesion size (mm)	Infection rate (%)	Lesion size (mm)
Conidia				
S1	30	4 b	100	9 bc
S2	30	2 b	100	4 e
S3	0	1 d	100	6 de
Chlamydospore				
B1	50	4 b	90	7 cd
B2	0	0 d	70	4 ef
B3	20	1 d	100	8 cd
B4	30	2 c	100	6 de
B5	0	0 d	100	4 e
B6	0	0 d	60	1 f
B7	10	1 d	100	9 bc
B8	0	0 d	80	8 bc
B9	0	0 d	100	4 e
B10	0	0 d	100	12 ab
Original isolate	0	0 d	80	9 bc
Medium (control)	100	13 a	100	14 a

^a^Four leaves were treated with a liquid culture on the upper side daily for 3 days before inoculation on the 4th day on two upper leaves and two lower leaves.

^b^Values followed by the same letter in the same column are not significantly different using Fisher’s least significant difference test at *P* = 0.05.

### Isolation of resistance-activating substances

When a culture of *H. phialophoroides* KVF-2 B6 was separated into supernatant and mycelium mat by centrifugation, the supernatant was not very effective in activating disease resistance on pepper leaves against *P. capsici*. The lesion size on the untreated lower leaves was not much different from that of control (Table 3). On the other hand, mycelium in suspension subjected to ultrasonification or mycelium mat ground in liquid nitrogen was very effective in activating disease resistance, reducing the lesion size on untreated lower leaves from 11 mm in diameter in the control to 2 to 4 mm. The result suggests that the resistance-activating substances were retained mainly in the mycelium after production. Therefore, for extraction of the resistance-activating substances, mycelium mat was ground with liquid nitrogen and the resulting powder was extracted separately with various solvents.

**Table 3:** **Comparison of ability of supernatant and mycelium pellet of liquid culture of** ***Humicola phialophoroides*** **KVF-2 B6 to reduce disease incidence of Phytophthora blight of pepper caused by** ***Phytophthora capsici*** ^**a**^

Treatment	Inoculation site^b^			
	Upper leaf surface (treated surface)		Lower leaf surface (non-treated surface)	
	Infection rate (%)	Lesion size (mm)	Infection rate (%)	Lesion size (mm)
Original culture	0	0 c	90	6 b
Supernatant	50	7 b	70	9 b
Mycelium pellet				
Sonification	30	4 bc	30	4 c
Grinding	10	1 c	30	2 c
Liquid medium (control)	100	15 a	80	11 a

^a^Four leaves were treated with a liquid culture on the upper side daily for 3 days before inoculation on the 4th day on two upper leaves and two lower leaves.

^b^Values followed by the same letter in the same column are not significantly different using Fisher’s least significant difference test at *P* = 0.05.

When mycelium powder was extracted with water, 50% ethanol or 95% ethanol, the extracts were able to prevent or minimize infection of *P. capsici* on treated upper pepper leaves. The 50% ethanol extract was also capable of reducing the lesion diameter on the lower untreated leaf surface from 13 mm in the control to 3 mm (Table 4). Methanol, acetone or hexane extract was also able to reduce the infection rate from 90% in the control to 30 to 60% and lesion size from 12 mm to 3 to 6 mm on the treated upper leaves. These extracts were essentially ineffective in reducing the infection rate and lesion size on the untreated lower leaves. Based on these results, 50% ethanol was selected for use in the extraction of resistance-activating substances from the mycelium powder in the subsequent study.

**Table 4:** **Effectiveness of different solvents to extract substances, from mycelium powder of** ***Humicola phialophoroides*** **KVF-2 B6, capable of reducing disease incidence of Phytophthora blight of pepper caused by** ***Phytophthora capsici*** ^**a**^

Solvent	Inoculation site			
	Upper leaf surface (treated surface)		Lower leaf surface (non-treated surface)	
	Infection rate (%)	Lesion size (mm)	Infection rate (%)	Lesion size (mm)
Water	10	1	30	5
50% ethanol	0	0	50	3
95% ethanol	0	0	50	7
Methanol	60	6	90	13
Acetone	30	3	80	10
Hexan	50	5	70	10
Medium (water)	90	12	100	13

^a^Four leaves were treated with a liquid culture on the upper side daily for 3 days before inoculation on the 4th day on two upper leaves and two lower leaves.

### Biological activity of the resistance-activating substances

Mycelium extract of *H. phialophoroides* was not inhibitory to germination of zoospores of *P. capsici* either on glass slide or directly on pepper leaves. It was even slightly stimulatory to germination on pepper leaves, increasing the germination from 70% in the control to 92% (Table 5). The extract was also stimulatory to germ tube growth, increasing the germ tube length in 6 h from 84 μm in the control to 182 μm on a glass slide and from 208 μm to 255 μm on a pepper leaf. The result shows that the inability of *P. capsici* to infect the upper pepper leaves treated with the extract was also due to activation of host resistance rather than inhibition of the pathogen. Therefore, for assaying the resistance activation activity only the upper treated leaves were used in the subsequent study.

**Table 5:** **Effect of mycelium extract of** ***Humicola phialophoroides*** **KVF-2 B6 on germination of zoospores of** ***Phytophthora capsici*** **on glass slides or pepper leaves** ^**a**^

Treatment	Germination (%)	Germ tubes length (μm/6 h)
On glass slide		
Mycelium extract	93 a	182 c
Water (control)	87 a	84 d
On pepper leaf		
Mycelium extract	92 a	255 a
Water (control)	70 b	208 b

^a^Values followed by the same letter in the same column are not significantly different using Fisher’s least significant difference test at *P* = 0.05.

To determine the strength of the resistance activated by the extract, each extract-treated circle was inoculated with 5, 10, 20, or 40 zoospores of *P. capsici*. The result showed that the activated resistance was able to effectively withstand the infection potential imposed by 40 zoospores/test circle, the highest concentration tested. When the zoospore concentration was increased from the original 5 to 40 zoospores/test circle, the infection rate was increased only from 0 to 20% in comparison with 100% in the control, and the lesion diameter was increased only from 0 to 2 mm in comparison with 15 mm in the control (Table 6).

**Table 6:** **Effect of zoospore concentration of** ***Phytophthora capsici*** **on resistance activated by the mycelium extract of** ***Humicola phialophoroides*** **KVF-2 B6 on pepper leaves** ^**a**^

Zoospre concentration (no./test circle)	Infection rate (%)	Lesion size (mm)^b^
5 (original)	0	0 c
10	20	2 b
20	20	1 b
40	20	2 b
Control (water)	100	15 a

^a^Extract was spread on five test circles on each of the two leaves three times within 1 h and inoculation was performed on each circle after 24 h.

^b^Values followed by the same letter are not significantly different using Fisher’s least significant difference test at *P* = 0.05.

To determine how long after extract application pepper leaves will become resistant, test circles on leaves were inoculated with zoospores of *P. capsici* 0, 2, 8, 16, or 32 h after extract application. When inoculated 0 or 2 h after extract application, pepper leaves were not resistant to *P. capsici*. However, 4 h or more after extract application, all the treated leaves became resistant to *P. capsici*, reducing the infection rate from 90% in the control to 10 to 20%, and lesion diameter from 10 mm to 1 to 2 mm (Table 7).

**Table 7:** **Time required for development of resistance on pepper leaves against** ***Phytophthora capsici*** **induced by the mycelium extract of** ***Humicola phialophoroides*** **KVF-2 B6** ^**a**^

Inoculation time after extract treatment (h)	Infection rate (%)	Lesion size (mm)^b^
0	80	10 a
2	80	9 a
4	20	2 b
8	20	1 b
16	10	2 b
32	10	2 b
Control (water)	90	10 a

^a^Extract was spread on five test circles on each of the two leaves three times within 1 h and inoculation was performed on each circle after 24 h.

^b^Values followed by the same letter are not significantly different using Fisher’s least significant difference test at *P* = 0.05.

Since zoospores do not infect leaves right away after inoculation, the time required for infection was also determined for estimation of time needed for development of resistance. Each pepper leaf was inoculated with five 2-μl drops of a zoospore suspension of *P. capsici* along the edge of the leaf and a 10-μl drop of molten agar was added to each inoculation drop to fix the inoculum on the target site. The solidified agar drops with zoospores attached were removed at various time intervals. When inoculum was removed 0 to 4 h after inoculation, no infection occurred. However, when inoculum was removed 8 to 32 h after inoculation, infection rate was 100% and the lesion size was the same as that of the control without inoculum removed for every interval tested (Table 8).

**Table 8:** **Time required for zoospores of** ***Phytophthora capsici*** **to infect pepper leaves after inoculation**

Time of inoculums removal after inoculation (h)	Infection rate (%)	Lesion size (mm)
0	0	0
2	0	0
4	0	0
8	100	15
16	100	15
32	100	15
Control (water)	100	15

To determine the effect of water washing on resistance activity after resistance activation, treated pepper leaves were each sprayed with 20 ml distilled water with an atomizer 24 h after extract treatment and inoculated with zoospores of *P. capsici* after air drying. After resistance activation, washing with water did not have much effect on resistance activity. The infection rate was reduced from 100% in the control to 30% with washing in comparison with 10% without washing, while the lesion diameter was reduced from 15 mm in the control to 2 mm with washing and 1 mm without washing (Table 9). The infection rate and lesion size on pepper leaves treated with extract, washed with water as soon as the extract had dried, and inoculated with zoospores 24 h later were the same as those on the non-treated control.

**Table 9:** **Effect of water washing on resistance of pepper leaves against** ***Phytophthora capsici*** **induced by the mycelium extract of** ***Humicola phialophoroides*** **KVF-2 B6** ^**a**^

Treatment	Infection rate (%)	Lesion size (mm)^b^
Washing 24 h after treatment	30	2 b
No washing after treatment	10	1 b
Washing right after treatment	100	15 a
No treatment (control)	100	15 a

^a^Extract was spread on five test circles on each of the two leaves three times within 1 h and inoculation was performed on each circle after 24 h.

^b^Values followed by the same letter are not significantly different using Fisher’s least significant difference test at *P* = 0.05.

### Direction and distance of the resistance activation

**Figure 1:**
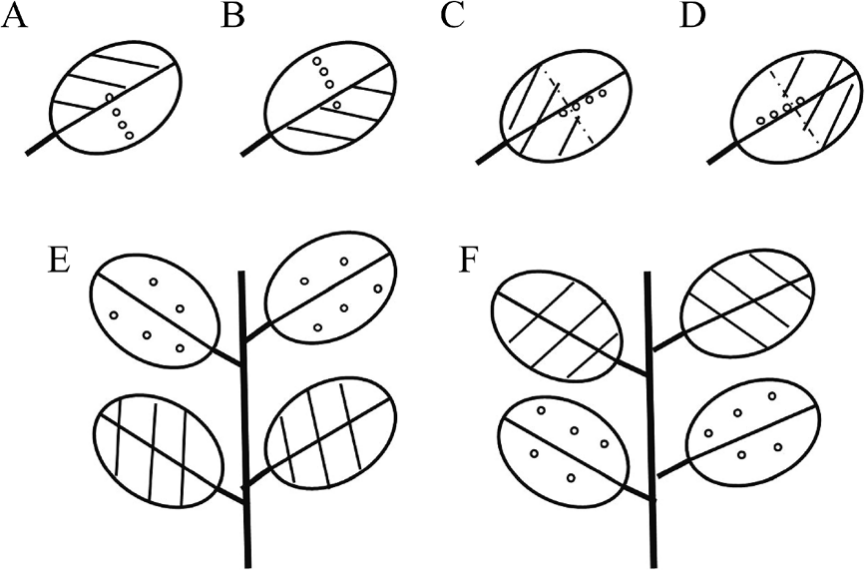
**Diagrammatic sketch showing designs for testing movement direction and distance of the resistance activity against** ***Phytophthora capsici*** **on pepper activated by mycelium extract of** ***Humicola phialophoroides*** **KVF-2 B6.** Parallel lines represent treatment with mycelium extract, and circles represent inoculation sites. Movement **(A)** from left to right; **(B)** from right to left; **(C)** from base to terminal; **(D)** from terminal to base; **(E)** from lower leaves to upper leaves; **(F)** from upper leaves to lower leaves.

To study sideward movement of the resistance-activating substances, mycelium extract was applied to the left or right half of pepper leaves and inoculated with zoospores of *P. capsici* 0, 5, 10, 15 mm away from the treated area (Figure 1). The result showed that the resistance-activating phenomenon was not able to move sideward across the midrib. Although infection was prevented in the area treated with the extract, infection rate was 100% and lesion diameter was 13 to 14 mm just 5 mm away from the treated area (Table 10). The mycelium extract was also applied to the terminal or basal half of leaf surfaces and zoospores of *P. capsici* were used to inoculate the leaves 0, 5, 10 or 15 mm away from the treated area to determine whether the resistance-activating substances can move toward the apex and/or the base. The resistance-activating substances were able to move 5 mm toward the apex and 10 mm toward the base (Table 10). When the upper or lower two pepper leaves were treated with mycelium extract and both treated and non-treated leaves were inoculated with zoospores of *P. capsici*, only the treated leaves showed disease resistance (Table 10), indicating that the resistance-activating substances were not able to move to lower or upper leaf surfaces.

**Table 10:** **Movement direction and distance of the resistance activity against** ***Phytophthora capsici*** **on pepper activated by the mycelium extract of** ***Humicola phialophoroides*** **KVF-2 B6**

Position of leaf		Distance (mm)^a^	Infection rate (%)^b^	Lesion size (mm)^c^	Diagram (Figure1)
Extract application	Inoculation				
Left half	Right half	0	0	0 b^d^	A
		5	100	14 a	
		10	100	15 a	
		15	100	15 a	
Right half	Left half	0	0	0 b	B
		5	100	13 a	
		10	100	15 a	
		15	100	15 a	
Terminal half	Basal half	0	13	1 c	C
		5	63	6 b	
		10	88	13 a	
		15	88	13 a	
Basal half	Terminal half	0	13	1 d	D
		5	38	3 c	
Terminal half	Basal half	10	63	6 b	
		15	75	11 a	
Upper two leaves	Lower two leaves	0	0	0 b	E
		30	100	15 a	
Lower two leaves	Upper two leaves	0	0	0 b	F
		30	100	15 a	

^a^Distance between site of treatment and inoculation.

^b^Eight inoculation points were used for each treatment.

^c^Data were means of eight measurements.

^d^Values followed by the same letter in the same experimented design are not significantly different using Fisher’s test significant different test *P* = 0.05.

### Characteristics of the resistance-activating substances

The resistance-activating substances were very stable under high temperature. After exposure to 100°C in water bath for 20 min, the extract was able to reduce the lesion size caused by *P. capsici* on pepper leaves from 15 mm in diameter in the control to 2 mm. Even after autoclaving for 20 min, the extract was still capable of reducing the lesion diameter from 15 mm to 4 mm.

When the pH of the mycelium extract was adjusted from the original 6.7 to 4 or 10, its resistance activation ability remained about the same. The lesion size was reduced from 11 mm in diameter caused by *P. capsici* on the control leaves to 0 to 2 mm when pepper leaves were treated with the extract at three pH values before inoculation with the pathogen. The resistance-activating substances in the mycelium extract were relatively unstable at extreme pH values. When pH of the extract was adjusted to 2 or 12 for 24 h and then adjusted back to the original 6.7, its ability to reduce the lesion size on pepper leaves caused by *P. capsici* was decreased. The lesion diameter developed on the treated leaves was increased from 0 without treatment to 7 and 4 mm after the extract was exposed to pH 2 and 12, respectively.

The resistance activation activity of the mycelium extract was not affected by treatment with cation or anion exchange resins (Table 11). However, after treatment with activated charcoal, the resistance activation activity of the extract was greatly reduced. The lesion diameter developed on pepper leaves was increased from 2 mm spread with extract before treatment to 7 mm after treatment with activated charcoal.

**Table 11:** **Effects of adsorptive materials on the resistance activation activity of mycelium extract of** ***Humicola phialophoroides*** **KVF-2 B6 against Phytophthora blight of pepper caused by** ***Phytophthora capsici*** ^**a**^

Treatment	Infection rate (%)	Lesion size (mm)
Before treatment	20	2
Cation extract resins	0	0
Anion extract resins	0	0
Activated charcoal	60	7
Water (control)	100	12

^a^Extract was spread on five test circles on each of the two leaves three times within 1 h and inoculation was performed on each circle after 24 h.

When mycelium extract was fractionated by chromatography on a silica gel column, fractions 1, 3 and 4 were able to completely prevent the disease development of Phytophthora blight on pepper leaves (Table 12). Even fractions 2 and 5 were also capable of reducing the disease incidence to about 50% and lesion size more than 50% of the control.

**Table 12:** **Resistance activation ability of fractions 1 to 5 from silica gel column chromatography of mycelium extract of** ***Humicola phialophoroides*** **KVF-2 B6 eluted with a mixture of hexane and acetone (8:2), against Phytophthora blight of pepper caused by** ***Phytophthora capsici*** ^**a**^

Fraction no.	Infection rate (%)	Lesion size (mm)
1	0	0
2	30	5
3	0	0
4	0	0
5	70	7
Water (control)	100	15

^a^Each eluent was spread on five test circles on each of the two leaves three times within 1 h and inoculation was performed on each circle after 24 h.

## Discussion

Using the method developed in this study, 3 out of 121 isolates of microorganisms obtained from soils showed ability to produce substances capable of activating resistance in pepper leaves against infection by *P. capsici*. The results support the hypothesis that soil is an ideal place to search for microorganisms capable of producing resistance-activating substances. The technique may be useful for screening soil microorganisms for production of resistance-activating substances against other plant pathogens. This study also demonstrated the possibility of obtaining a better producer of resistance-activating substances through selection from the asexual population. Both single-conidium and single-chlamydospore progenies of *H. phialophoroides* displayed variation in ability to produce resistance-activating substances. This may suggest that the fungus is either a heterokaryon or a heteroplasmon (Burnett,^1975^).

The liquid culture of *H. phialophoroides* used in the initial resistance activation study consisted of the organism and the substances produced by it. However, in the subsequent study, ethanol extract of the mycelium powder was found to be as effective as the culture in resistance activation. Moreover, the resistance-activating ability of the extract was not reduced by exposure to 100°C for 20 min or autoclaving for 20 min, indicating that only chemical substances were involved in resistance activation.

The movement distance of the resistance-activating substances produced by *H. phialophoroides* appears to be relatively short. In addition to being able to move from the upper leaf surface to the lower leaf surface, the resistance-activating phenomenon was capable of moving 5 mm acropetally and 10 mm basipetally in leaves. However, they were not able to move sideward through the main veins, nor were they able to move to other leaves.

Pepper leaves became resistant to *P. capsici* only 4 h or more after treatment with mycelium extract of *H. phialophoroides*. Since 8 h was needed for zoospores of *P. capsici* to infect pepper leaves (Table 8), a total of about 12 h apparently was required for activation of resistance. In cucumber, resistance against *Colletotrichum lagenarium* was detected within 6 h after inoculation of the inducer *Pseudomonas syringae* pv. *syringae* (Smith et al.,^1991^). Tobacco leaves required about 2 weeks to develop resistance against *Peronospora hyoscyami* f. sp. *tabacina* following stem inoculation with the same organism (Cohen and Kuc,^1981^). Activation of resistance against tomato spotted wilt virus in tobacco was observed within 2 days after treatment with acibenzolar-S-methyl (Mandal et al.,^2008^).

The mechanism of resistance activation by the compounds produced by *H. phialophoroides* remains to be investigated. Although systemic acquired resistance commonly results from infection with a necrogenic pathogen, similar plant responses can also be triggered by certain natural and synthetic chemical compounds (Kessmann et al.,^1994^; Durrant and Dong,^2004^). Future study of plant responses to the resistance-activating substances found in this study will likely facilitate the elucidation of their resistance activation mechanism.

When mycelium powder of *H. phialophoroides* was extracted with water or ethanol, the resistance activation activity of the extracts was very strong. However, when it was extracted with methanol, acetone or hexane, the extracts showed some resistance activation ability, albeit the activity was relatively low. The result suggests that although the majority of the resistance activation components in the extract is hydrophilic, a small amount of the resistance activation components is hydrophobic. The bioassay result of the fractions from the silica gel chromatography suggests that the extract probably contains at least three compounds capable of activating host resistance. The resistance activation ability of the extract was not affected by treatment with either cation or anion exchange resins, indicating that none of the active components have positive or negative charge on their molecules.

Previously, treatment of pepper seeds and roots with *Trichoderma harzianum* was reported to be effective in reducing stem necrosis caused by *P. capsici* (Lyon and Newton,^1999^). Accumulation of the fungitoxic compound capsidiol was considered one of the contributing factors in delaying lesion development on stems of pepper plants (Ahmed et al.,^2000^). Fluorescent pseudomonas isolated from rhizospheres of pepper plants were found able to control Phytophthora blight of pepper by soaking seeds in the bacterial suspension before planting in the greenhouse (Rajkumar et al.,^2005^). Antagonistic bacteria isolated from rhizospheres of pepper, cucumber and tomato plants also were shown to be capable of reducing Phytophthora blight of pepper by drench or root-dip treatments with bacterial suspension in the field (Sang et al.,^2008^). In this study, the resistance-activating substances produced by *H. phialophoroides* were found to be stable under high temperature and were active at pH ranging from 4 to 10, indicating that they are suitable for development into a commercial product for control of Phytophthora blight of pepper and possibly other plant diseases as well. The chemical structures of these compounds and their disease control efficacy under field conditions and toxicity to the non-target organisms in nature remain to be investigated.

## Conclusion

Results show that *H. phialophoroides* is capable of producing multiple resistance-activating substances for control of Phytophthora blight of pepper. The substances are mostly retained in the mycelium. The study also indicated that none of the active components have positive or negative charges on their molecules.

## Authors’ original submitted files for images

Below are the links to the authors’ original submitted files for images. Authors’ original file for figure 1
