# Supplementary figures and images for: Multiple resistance-activating substances produced by Humicola phialophoroides isolated from soil for control of Phytophthora blight of pepper
Source: Bot Stud. 2014 Sep 11;55:40. doi: 10.1186/1999-3110-55-40 (PMC5432772; doi:10.1186/1999-3110-55-40)

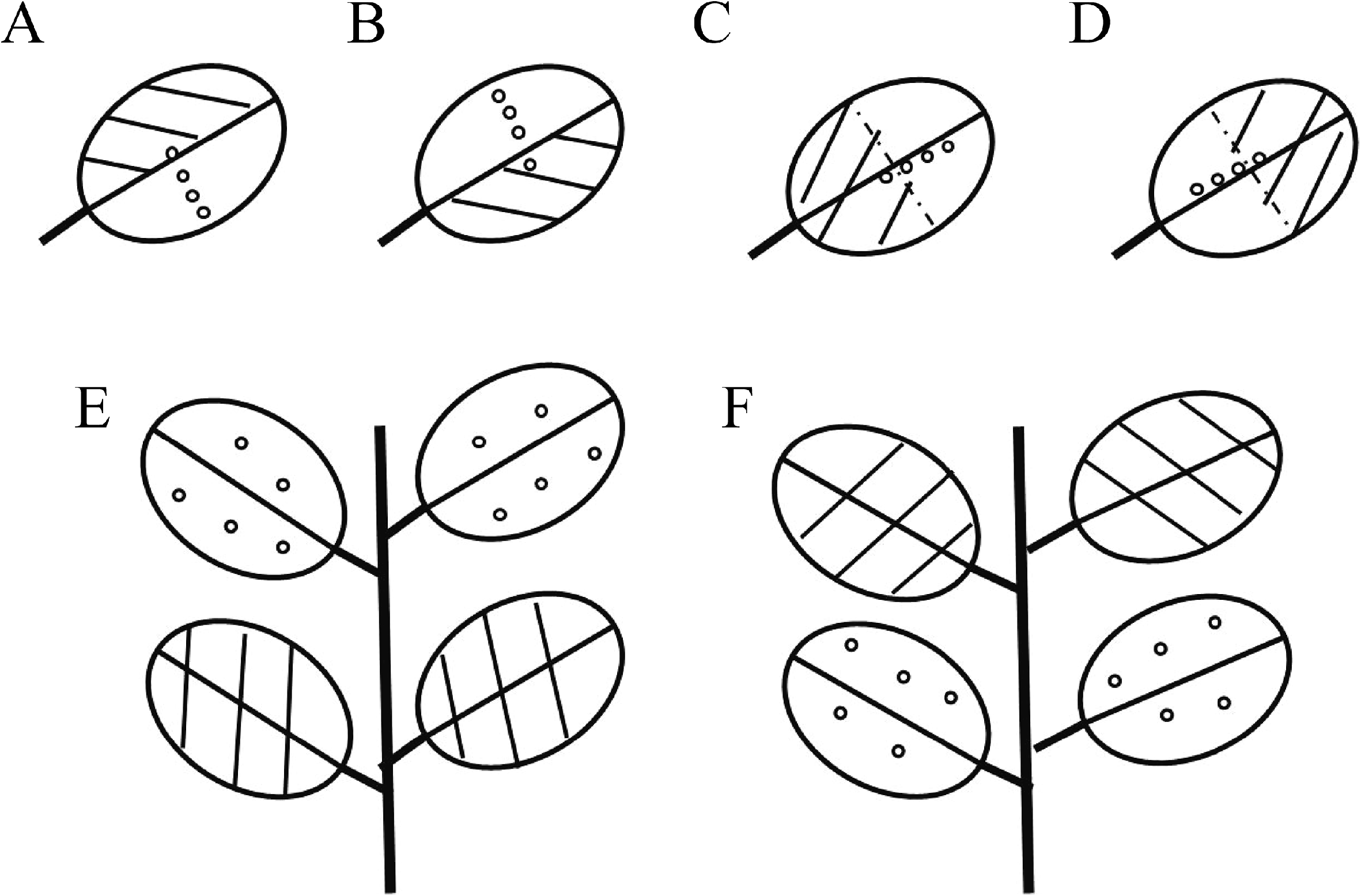

Supplement: Supplementary file 1 — Authors’ original file for figure 1 [file 40529_2012_92_MOESM1_ESM.tiff]
